# Treatment-responsive case of focal clivus IgG4-related hypertrophic pachymeningitis mimicking meningioma; case report

**DOI:** 10.1007/s13760-021-01667-5

**Published:** 2021-04-08

**Authors:** Shun Yamamuro, Hiroshi Negishi, Katsunori Shijo, Atsuo Yoshino

**Affiliations:** Department of Neurological Surgery, Nihon University School of Medicine, 30-1 Oyaguchi-Kamichou, Itabashi-ku, Tokyo, 173-8610 Japan

Dear Editor,

Sir, we report here a rare case of IgG4-related hypertrophic pachymeningitis which formed a symmetrical focal mass lesion in the clivus region.

**Fig. 1:**
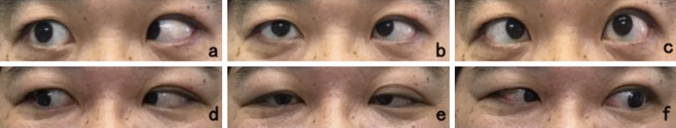
**a–c** Eye movement findings before surgery (**a** right gaze, **b** primary possession, **c** left gaze). **d–f** Eye movement findings after glucocorticoid treatment (**d** right gaze, **e** primary possession, **f** left gaze)

**Fig. 2:**
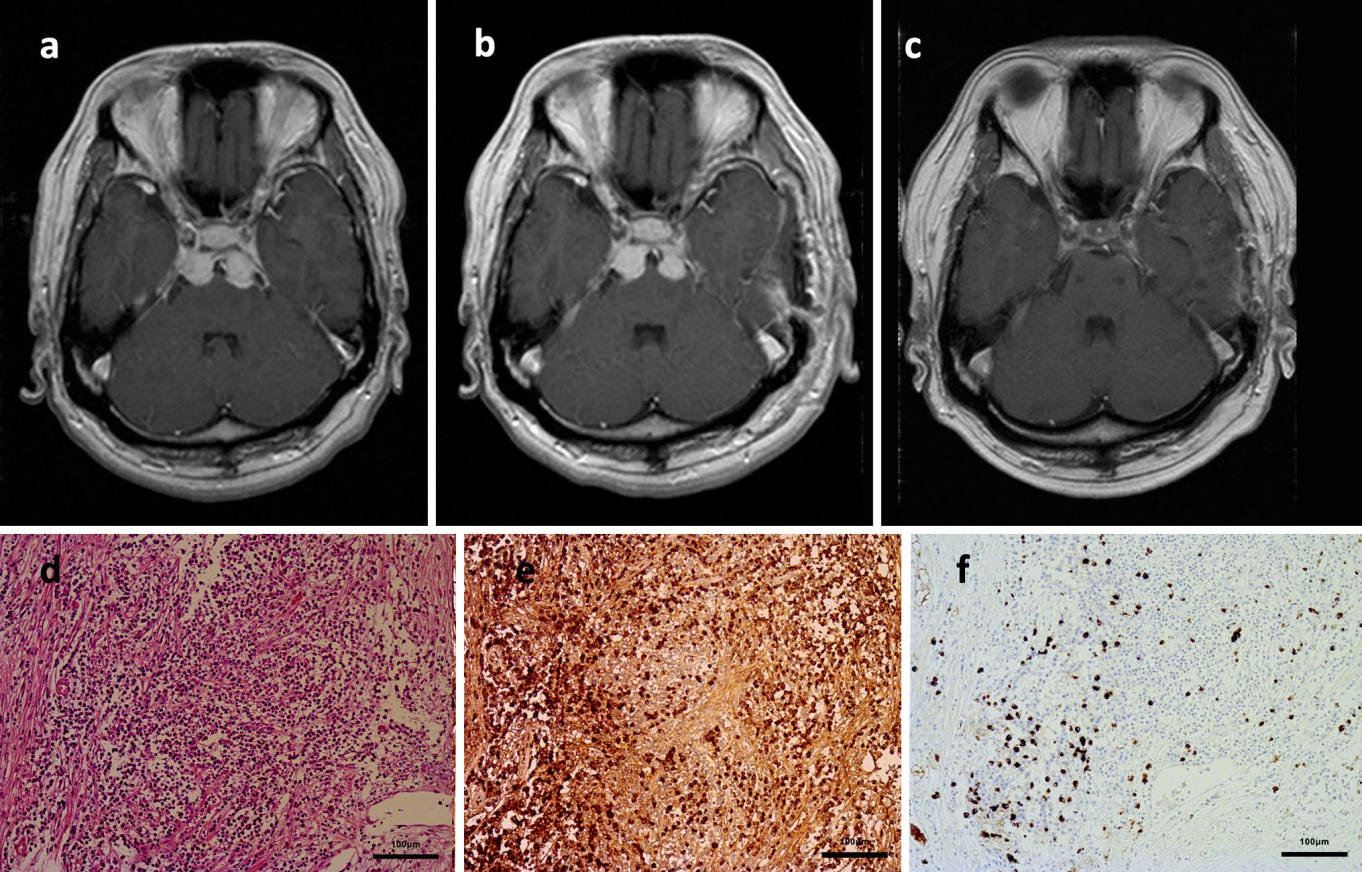
**a** Preoperative axial gadolinium-enhanced T1-weighted MR imaging. **b** Postoperative axial gadolinium-enhanced T1-weighted MR imaging. **c** Post-glucocorticoid treatment axial gadolinium-enhanced T1-weighted MR imaging). **d** Pathological findings via hematoxylin and eosin staining (original magnification × 100). **e, f** Pathological findings via immunohistochemical staining of IgG (**e** original magnification × 100) and IgG4 (**f** original magnification × 50)

A 51-year-old male was admitted to our hospital complaining of eye movement disorder. His consciousness level was clear and neurological examinations revealed no abnormalities except for bilateral abducens nerve palsies (Fig. 1a–c). He was engaged in general desk work and had no occupational and/or medical history which could be a risk factor for any disease. Magnetic resonance (MR) imaging disclosed a symmetrical mass lesion in the clivus region, which displayed iso-intensity on T1-weighted MR imaging, and low-intensity on T2-weighted MR imaging; also, homogenous enhancement was evident after contrast medium administration, and the mass lesion retracted the patient’s pons (Fig. 2a). Cerebral angiography revealed arterial supply via the bilateral meningohypophyseal trunk. Laboratory investigations including those for tumor markers did not yield any abnormal values, and no neoplastic lesions were found in other organs on computed tomography scans. The lesion was preoperatively diagnosed as a clivus meningioma.

The patient underwent surgical removal via a left-side anterior petrosal approach to remove the left-side mass. The intraoperative findings indicated that the mass was situated between the abducens nerve and pons. Removal of the mass proved very difficult because the tumor was very hard and tightly attached to the pons. To make a diagnosis and to avoid critical complications, the mass was removed partially. Pathological examinations including immunohistochemical staining demonstrated the presence of abundant IgG positive plasma cells resting on fibrous tissue. These cells also exhibited positive expression of IgG4, and the ratio was 15.2% of the positive expression of IgG (Fig. 2d–f). The above pathological findings were consistent with IgG4-related hypertrophic pachymeningitis.

The patient’s postoperative course was uneventful, and postoperative MR imaging demonstrated residual majority of the mass lesion (Fig. 2b). The peripheral blood IgG (885 mg/dl; reference range 861–1747 mg/dl) and IgG4 (66.7 mg/dl; reference range 4.8–105 mg/dl) were estimated postoperatively, and these levels were normal. Furthermore, evaluations of the peripheral blood PR3-ANCA, MPO-ANCA, anti-nuclear antibody, dsDNA, C3, C4, CH50, soluble interleukin-2 receptor, CD4 and CD8 were made; however, all of the values were negative or within the normal range. The patient underwent glucocorticoid treatment. Prednisolone was started at 60 mg/day to finish by tapering every 14 days after 1000 mg methylprednisolone for 3 days (the total glucocorticoid treatment period was 87 days). The patient’s bilateral abducens nerve palsies were particularly improved after the glucocorticoid treatment (Fig. 1d–f). The mass lesion disappeared completely on post glucocorticoid treatment MR imaging (Fig. 2c), and recurrence has not been recognized even though more than 5 years have passed without any further medication including glucocorticoid.

IgG4-related hypertrophic pachymeningitis is being increasingly recognized, but a consensus concerning the optimal diagnostic approach and therapeutic approach remains lacking [1]. Hypertrophic pachymeningitis usually exhibits a diffusely invasive pattern, but rarely demonstrates a focal mass as in the present case or in a few other case reports [2–5]. When hypertrophic pachymeningitis reveals a focal mass, it is difficult to distinguish from a tumor, such as meningioma and/or solitary fibrous tumor, preoperatively [2, 4, 5]. Tang et al. reported a case of clivus massive IgG4-related disease similar to the present case [5]. In their report, the mass was preoperatively diagnosed as meningioma, similarly to the present case, and both the postoperatively evaluated peripheral blood IgG and IgG4 were within the reference range [5]. Glucocorticoid treatment was not performed in their case since the mass was soft and totally removed via surgery; and the follow-up period was not described [5]. The effectiveness of glucocorticoid treatment for IgG4-related hypertrophic pachymeningitis has been noted in previous case reports [3, 4], and we also obtained a favorable outcome with remarkable shrinkage of the mass lesion and improvement of the palsies of the bilateral abducens nerves following glucocorticoid treatment. It is important therefore to perform surgery with the primary purpose of diagnosis to avoid complications, when we suspect IgG4-related disease for a skull base mass since it displays a good reaction for glucocorticoid treatment and in some cases, like the present one, can be completely cured. Biopsy surgery is a satisfactory treatment strategy for IgG4-related disease, which is suspected preoperatively or diagnosed intraoperatively, and full resection is not always necessary especially when the mass is located or adherent in a high-risk location and structure.
